# Myocardial functional recovery following durable ventricular assist device in children

**DOI:** 10.1016/j.jhlto.2024.100181

**Published:** 2024-11-17

**Authors:** Bhavikkumar Langanecha, Osami Honjo, Alyssa Power, Oshri Zaulan, Christoph Haller, Kristen George, Linda Fazari, Andrea Maurich, David Chiasson, Aamir Jeewa

**Affiliations:** a Labatt Family Heart Centre, Division of Pediatric Cardiology, Department of Pediatrics, University of Toronto, Hospital for Sick Children, Toronto, Ontario, Canada; b Division of Critical Care Medicine, Department of Pediatrics, University of Toronto, Hospital for Sick Children, Toronto, Ontario, Canada; c Labatt Family Heart Centre, Division of Cardiovascular Surgery, Department of Surgery, University of Toronto, The Hospital for Sick Children, Toronto, Ontario, Canada; d Department of Laboratory Medicine & Pathobiology, University of Toronto, Hospital for Sick Children, Toronto, Ontario, Canada

**Keywords:** left ventricular assist device (LVAD), myocardial functional recovery, ventricular assist device explant, heart failure, heart transplant, reverse remodeling

## Abstract

**Background:**

Ventricular assist device (VAD) explantation following myocardial functional recovery (MFR) for heart failure (HF) is uncommon in children and is associated with a risk of HF recurrence.

**Material and Methods:**

Retrospective, single-center study of pediatric patients who were supported with durable VADs, both intracorporeal continuous flow devices (CFD) and paracorporeal pulsatile flow devices (PFD), between 2004 and 2022.

**Results:**

A total of 74 children, of which 43 were female, underwent VAD implantation (PFD = 61 and CFD = 14) at a median (interquartile range) age of 5.6 (0.8, 13.5) years and with a weight of 16.2 (7.5, 40.7) kg. From this cohort, we identified 9 of 75 (12%) children who underwent VAD explantation for MFR. Of those, 7 of 9 (77%) were under 2 years of age and 6 of 9 (67%) were supported for >90 days. Five patients had dilated cardiomyopathy, 3 with anomalous left coronary artery from pulmonary artery and 1 with tachycardia-induced cardiomyopathy. Six were listed for transplantation as a part of their HF management strategy following VAD implantation. After explant, 8 of 9 patients remained in HF remission with no symptoms and stable left ventricular function. One patient had a recurrence of HF following explantation after demonstrating MFR while on VAD support.

**Conclusions:**

MFR resulting in VAD explantation is feasible in children with chronic HF especially for those <2 years of age. Further work is needed to better identify the features that promote MFR and maintain it after explant.

## Background

The use of durable ventricular assist devices (VAD) is increasing in the pediatric population with end-stage heart failure (HF), especially as a bridge to heart transplantation (HT).^1^ Mechanical circulatory support (MCS) on a VAD can lead to significant reverse remodeling with respect to the size, structure, cellular, extracellular, and molecular characteristics of the failing myocardium.^2^, ^3^, ^4^ The reverse remodeling leads to significant improvement in myocardial contractility and myocardial functional recovery (MFR) in a subset of patients.^5^, ^6^, ^7^ If possible, VAD explantation following MFR remains an attractive possibility, as HT is associated with its own complications.^8^ The rate of VAD explantation for MFR is low (1.2-5%) in adult registry data.^9^, ^10^ However, it is reported at a higher rate (40-73%) in prospective studies in adult patients with nonischemic cardiomyopathy with protocolized strategies for VAD management and guideline-directed medical therapy (GDMT).^5^, ^6^ Younger age and nonischemic cardiomyopathy are the common predictors of MFR in adults.^9^ VAD explantation following MFR in the pediatric population is rare.^1^ Meira et al reported the finding that myocarditis and children less than 2 years of age were predictors of VAD explantation following MFR.^11^ One of the major concerns for VAD explantation following MFR is the risk of recurrence of significant HF.^5^, ^12^, ^13^

Our aim was to report on our institutional experience of the incidence, clinical characteristics, and short- to intermediate-term outcomes of the children who underwent VAD explantation following MFR.

## Material and methods

### Study population

We performed a retrospective chart review of all patients younger than 18 years of age who underwent durable VAD implantation, defined as a paracorporeal pulsatile flow device (PFD) or intracorporeal continuous flow device (CFD), at the Hospital for Sick Children between 2004 and 2022. Patients who were only supported on temporary MCS, including extracorporeal membrane oxygenation and temporary CFDs, such as Rotaflow and Centrimag, were excluded. Clinical information was extracted from the medical record, including demographics, underlying cardiac disease, type and duration of mechanical support, echocardiographic findings, hemodynamic data, left ventricular (LV) histopathologic findings, and genetic information. The study was approved by the Research Ethics Board and informed consent was waived due to the retrospective nature of the study.

### Assessment and management pathway for MFR

**Figure 1:**
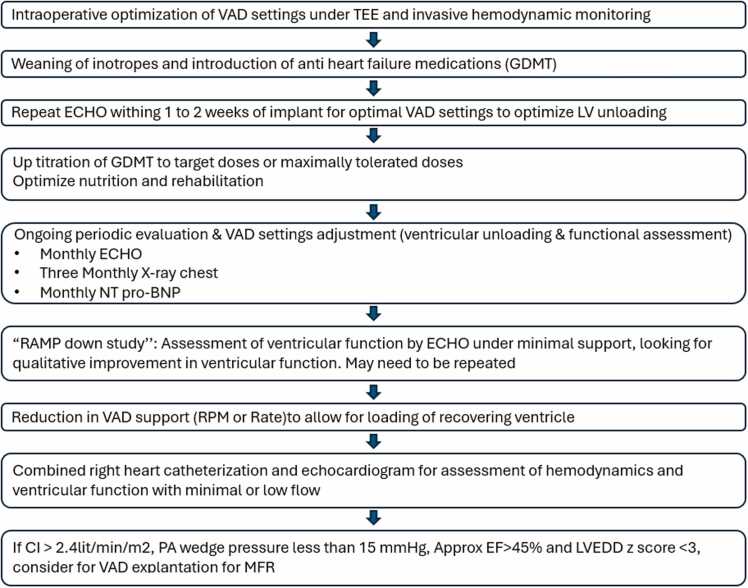
Flowchart for assessment and management plan for myocardial functional recovery on VAD. CI, cardiac index; ECHO, echocardiogram; EF, ejection fraction; GDMT, guideline-directed medical therapy; LVEDD, left ventricular end-diastolic diameter; MFR, myocardial functional recovery; NT-proBNP, N-terminal prohormone of brain natriuretic peptide; PA, pulmonary artery; RPM, revolutions per minute; TEE, transesophageal echocardiogram; VAD, ventricular assist device; X-ray, chest radiograph.

Figure 1 demonstrates our assessment and management pathway for MFR following durable VAD implantation. If there is persistent improvement in ventricular function with approximate ejection fraction more than 45% and LV end-diastolic dimension z-score less than 3 by echocardiogram during periodic assessment, then we assess myocardial function by combined cardiac catheterization and echocardiogram with VAD weaning under general anesthesia. Following baseline assessment, the support was gradually reduced every 15 minutes until minimal or no support. Hemodynamics and echo findings were obtained before each reduction in VAD support. If combined assessment reveals cardiac index more than 2.4 liter/min/m^2^ with pulmonary artery (PA) wedge pressure less than 15 mm Hg and approximate ejection fraction more than 45% with left ventricular end-diastolic diameter (LVEDD) z-score less than 3, then VAD explantation is considered. Following VAD explantation, patients are kept on maximally tolerated GDMT with periodic follow-up in the Heart Function clinic.

### Statistical analysis

Standard descriptive statistics were used to summarize baseline demographics and clinical variables. Continuous variables are presented as median ± interquartile range (IQR). Dichotomous and polychotomous variables were summarized by frequencies. Categorical variables were analyzed using Fisher’s exact test and continuous variables analyzed using Mann-Whitney U test between 2 groups. Kaplan-Meier survival method with Log-rank test was used to compare VAD explant for MFR between age less than 2 years and more than 2 years.

### Theory

VAD explantation for MFR is an attractive outcome but remains rare and is associated with the risk of recurrence of HF. The clinical characteristics and post-VAD explant outcomes for MFR are not well studied in children. This single-center study may provide the understanding of clinical characteristics and outcomes of VAD explant in children and open the pathway for further studies to identify the predictors of potential MFR following VAD in children.

## Results

**Figure 2:**
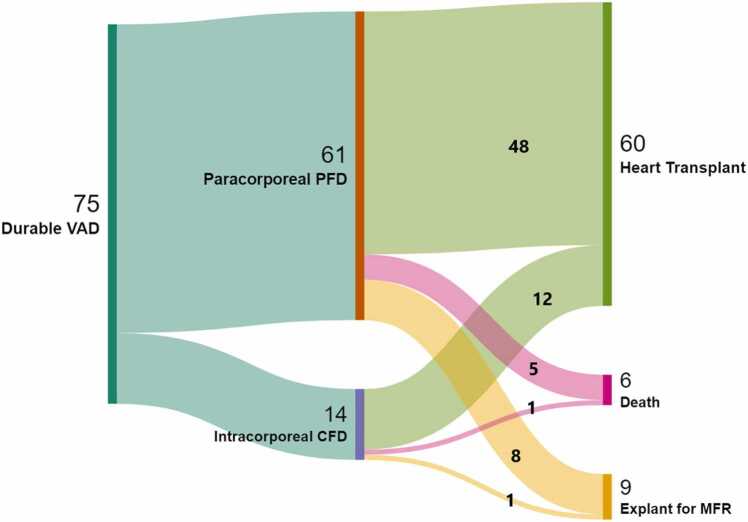
Sankey diagram: outcomes following VAD implantation. CFD, continuous flow devices; PFD, pulsatile flow devices; MFR, myocardial functional recovery; VAD, ventricular assist device.

During the study period, a total of 74 patients underwent durable VAD implantation for end-stage HF, including 1 patient who underwent VAD implantation twice for recurrent HF with severely reduced ventricular function following VAD explant for MFR. For analysis, we considered these 2 VAD implantations in the same patient as 2 separate events. Table 1 presents baseline demographics, diagnosis, and clinical characteristics. Patients underwent VAD implant at a median age of 5.6 years (IQR 0.81, 13.5) and with a weight of 16.2 kg (IQR 7.55, 40.75). At implantation, more than two-thirds of patients (51/75, 68%) were Interagency Registry for Mechanically Assisted Circulatory Support (INTERMACS) profile 2 and 20 patients (27%) were Interagency Registry for Mechanically Assisted Circulatory Support profile 1 including 12 patients on extracorporeal membrane oxygenation (ECMO) support. The most common underlying primary cardiac diagnosis was dilated cardiomyopathy (DCM)/myocarditis (63/75, 84%). Thirty out of 63 (48%) patients with DCM/myocarditis had a first presentation for their HF at the index hospitalization when the VAD implanted. The most commonly used devices were paracorporeal PFD, such as the Berlin Heart EXCOR. Sixty-one out of 75 (81%) patients were supported with paracorporeal PFD, out of which 23 patients were on biventricular assist device support. There were 14 patients (19%) who were supported with an intracorporeal CFD, either HeartWare HVAD or HeartMate 3. Patients were supported for a median duration of 57 days (IQR 22, 129.5). In the current era (2014-2023), the median duration of support was 111 days (IQR 59.5, 198) which further increased to 145 days (IQR 52, 193.5) in children less than 2 years. A total of 60 patients (80%) underwent successful HT, including 1 patient who required VAD explant for excessive clot burden in the pump, even after pump exchange, which was then shortly followed by a transplant. Six patients (8%) died while on VAD support (Table 2). Figure 2 describes the different outcomes following VAD implantation.

**Table 1:** Baseline Demographics, Clinical Characteristics of Whole Cohort and Comparision Between Explant and No-Explant Cohort.

	All (N = 75)	Explant for recovery (N = 9)	No explant (N = 66)	*p*-value
Age (years)	5.63 (0.81, 13.53)	0.6 (0.4, 1.2)	8.1 (1.1, 13.87)	0.003^a^
Sex				
Female	43 (57%)	5 (56%)	38 (58%)	0.9
Male	32 (43%)	4 (44%)	28 (42%)	
Pre ECMO	12 (16%)	2 (22%)	10 (15%)	0.6
INTERMACS profile				
1	20 (27%)	2 (22%)	18 (27%)	0.66
2	51 (68%)	6 (67%)	45 (68%)	
3	4 (5%)	1 (11%)	3 (5%)	
Diagnosis				
DCM/myocarditis^a^	63 (84%)	6 (67%)	57 (86%)	0.21
Other cardiomyopathy	2 (3%)	0 (0%)	2 (3%)	
CHD	10 (13%)	3 (33%)	7 (11%)	
Days of support	57 (22, 129.5)	148 (53, 174)	52 (20.25, 104)	0.04^a^
Type of device				>0.9
CFD	14 (19%)	1 (11%)	13 (20%)	
PFD	61 (8%)	8 (89%)	53 (80%)	

Abbreviations: CFD, continuous flow device; CHD, congenital heart disease; DCM, dilated cardiomyopathy; ECMO, extracorporeal membrane oxygenation; PFD, pulsatile flow device.

Data presented as median (interquartile range) or number (percentage).

a DCM/myocarditis includes tachycardia-induced cardiomyopathy patients.

**Table 3:** Demographics, Clinical, and ECHO Findings of VAD Explant Cohort at Time of VAD Implant

ID	Time of VAD implant			Sex	Diagnosis	Genetic testing	ECHO finding at VAD implant			
	Age (years)	Wt (Kg)	BSA				RVF	LVF	LVEF (%)	LVEDD z-score
1	1.2	9.8	0.51	M	ALCAPA	NA	Severely reduced	Severely reduced	10	8.9
2	0.4	9	0.47	M	DCM	VUS in CDH2	NA	Severely reduced	NA	7.2
3	1.0	7.6	0.42	F	Tachycardia-induced cardiomyopathy	NA	Moderately reduced	Severely reduced	17	9.6
4	0.3	4.6	0.28	F	DCM	Carrier of pathogenic mutation GAA (Pompe disease)	Good	Severely reduced	18	7.9
5	0.6	5.88	0.34	M	ALCAPA	NA	Good	Severely reduced	6	11.2
6	0.4	4.68	0.26	F	DCM	VUS in PKP2	Good	Severely reduced	10	7.1
7	0.4	6.29	0.33	F	ALCAPA	NA	Good	Severely reduced	17	8.3
8	11	35	1.2	M	DCM	VUS in RBM20	Severely reduced	Severely reduced	21	6.8
9	5.2	16	0.61	F	DCM	Likely Pathogenic Mutation TPM1	Severely reduced	Severely reduced	25	6.2

Abbreviations: ALCAPA, anomalous left coronary artery from pulmonary artery; BSA, body surface area; DCM, dilated cardiomyopathy; EF, ejection fraction in echocardiogram; LVEDD z-score, left ventricular end-diastolic diameter z-score; LVF, qualitative left ventricular function; NA, not available; RVF, qualitative right ventricular function; VUS, variants of unknown significance; Wt, weight.

### VAD explant cohort

From the overall cohort, 9 patients (12%) underwent VAD explantation following MFR. All patients except 1 (89%) were supported with paracorporeal PFD. The baseline demographics and echocardiogram findings before VAD implantation are outlined in Table 3. The underlying diagnosis was DCM in 5 (56%), and tachycardia-induced cardiomyopathy in 1 (11%). There were 3 patients (33%) with anomalous left coronary artery from the pulmonary artery (ALCAPA). All patients with ALCAPA underwent surgical coronary artery translocation before VAD implantation. One DCM patient underwent PA banding as an initial HF management due to prematurity and extremely small size before VAD implantation. Six patients were listed for HT as a part of their HF management strategies while on VAD support. All patients except 2 were <2 years of age at time of VAD implantation and 6 patients were supported with a VAD for more than 90 days. At the time of VAD explantation, 3 patients underwent additional cardiac surgical interventions. Two patients with DCM underwent PA banding at the time of VAD explantation, to improve ventricular-ventricular interactions, and 1 patient underwent mitral valve annuloplasty. Of note, no patient who had a sustained improvement in ventricular function on weaning echocardiogram failed the final combined cardiac catheterization and echocardiogram assessment for recovery in our cohort.

**Table 4:** Pathology Findings of LV Myocardium Core Samples Taken at Time of VAD Implantation

ID	Diagnosis	Pathology of LV sample		
		Myocardial fibrosis	EFE	Myocyte vacuolization
1	ALCAPA	Mild	No	No
2	DCM	No	Moderate	No
3	Tachycardia-induced CM	Mod	Severe	Yes
4	DCM	No	No	Yes
5	ALCAPA	Severe	No	Yes
6	DCM	No	NA	No
7	ALCAPA	Mild	NA	No
8	DCM	Mild	Severe	Yes
9^a^	DCM	NA	NA	NA

Abbreviations: ALCAPA, anomalous left coronary artery from pulmonary artery; CM, cardiomyopathy; DCM, dilated cardiomyopathy; EFE, endocardial fibroelastosis; HF, heart failure; MFR, myocardial functional recovery; LV, left ventricular; VAD, ventricular assist device.

a Patient with recurrence of HF following VAD explantation for MFR and had no LV biopsy sample at time of implant.

The histopathological findings of core myocardial samples taken at time of implantation were variable in severity with respect to myocardial fibrosis, endocardial fibroelastosis, and myocyte vacuolization. There was either no or mild myocardial fibrosis seen in the DCM LV biopsy samples, with only 1 (ALCAPA) biopsy showing severe fibrosis. No patient had evidence of active inflammation suggestive of myocarditis (Table 4)

**Table 5:** Echo and Hemodynamics Findings at VAD Weaning and Post Explant Follow-Up

ID	Duration of VAD support (days)	Listed for HTx	ECHO findings during VAD weaning						Hemodynamics during VAD weaning					Post explant follow-up			
			LVF	LVEF (%)	MR	RVF	TR	LVED Z-score	RAP	LAP/PCWP	CI	PAP	MVO2 (%)	F/up (years)	Last LVEF %	LVED Z-score	Ross/NYHA class
1	29	No	Mod	40	No	NA	Trace	1.2	11	13	NA	25	70	10.8	61	3.4	1
2	148	Yes	Mod	45	Mild	Good	Mod	2.1	6	NA	NA	NA	71	89.8	62	2.2	1
3	45	Yes	Mild	46	Trace	Good	No	2.6	NA	NA	NA	NA	67	9.8	51	−0.04	1
4^a^	174	Yes	Mild	50	Mild	Good	Trace	−1.2	4	6	NA	13	61^a^	23.1	50	1	1
5	207	Yes	Mild	62	Trace	Good	Trace	2.5	NA	8	4.5	13	71	1.6	60	2.5	1
6	148	Yes	Good	53	No	Good	Mild	2.9	4	6	2.96	17	59	1.5	63	3.1	1
7	53	No	Mild	44	No	Good	Mild	2.5	5	8	2.92	14	60	1.6	58	3.4	1
8	580	No	Good	60	Sever	Good	Mild	2	4	10	2.8	19	68	0.8	30-35	4.9	1
9^a^	111	Yes	Good	52	NA	Good	NA	0.8	5	5	NA	NA	53	NA^b^	NA^b^	NA^b^	NA^b^

Abbreviations: CI, cardiac index (liter/min/m^2^); ECHO, echocardiogram; HTx, heart transplant; LAP, left atrial pressure; LVEDD, left ventricular end-diastolic diameter; LVF, qualitative left ventricular systolic function; LVEF, left ventricular ejection fraction; MR, mitral regurgitation; PAP, mean pulmonary artery pressure; NYHA, New York Heart Association; NA, not available; PCWP, pulmonary capillary wedge pressure; RAP, right atrial pressure; RVF, qualitative right ventricular systolic function; TR, tricuspid valve regurgitation; VAD; ventricular assist device.

a Hemodynamic assessment by cardiac catheterization and ECHO on separate occasions.

b Post heart transplant.

One patient with DCM underwent VAD explant for MFR after 18 months of VAD support with a CFD. This patient presented with decompensated HF at the age of 11 years. Genetic testing revealed a variant of unknown significance, and the family did not wish to pursue assessment for heart transplantation. This patient developed ventricular functional recovery over the time and underwent VAD explant with mitral valve repair. At the last follow-up, this patient is now >10 months post explant and has a moderate LV dysfunction on GDMT, but is asymptomatic (New York Heart Association class 1).

Notably, 1 patient developed recurrence of HF following VAD explantation. This patient was 5 years old at time of initial VAD implantation with diagnosis of DCM due to likely pathogenic TPM1 gene mutation and was supported with a PFD for 111 days (patient number 9). Once evidence of MFR was demonstrated, the VAD was explanted, and the child was in HF remission for approximately 9 months. However, despite optimal medical management, there was recurrence of severe HF necessitating urgent VAD implantation, this time with a CFD. The patient was discharged from hospital on VAD support and was ultimately transplanted after 81 days of support. The genetic testing of remaining 4 patients with DCM who had a VAD explant for MFR showed variants of unknown significance.

**Figure 3:**
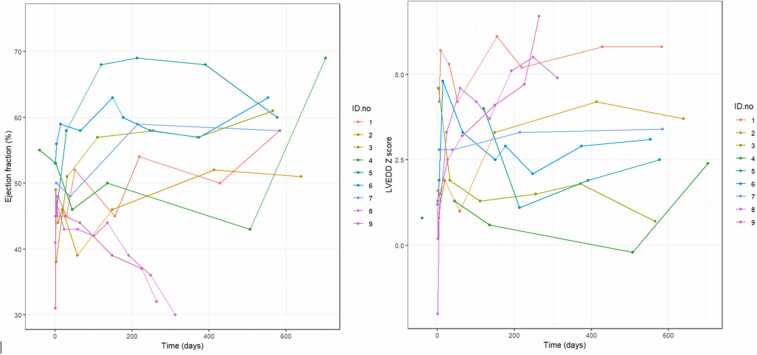
Trend of LV ejection fraction and LVEDD z-score for first 2 years following VAD explant. LV, left ventricular; LVEDD, left ventricular end-diastolic diameter; VAD, ventricular assist device.

The remaining VAD explant patients have a post explant median follow-up of 28.7 months (IQR 18.6, 120.1). All patients are asymptomatic (New York Heart Association/Ross Functional class 1) with stable ventricular function and without HF recurrence. Table 5 demonstrates echocardiographic and hemodynamic findings at VAD weaning and post explant. All patients except 1 with tachycardia-induced cardiomyopathy remain on angiotensin receptor/neprilysin inhibitor or angiotensin-converting enzyme inhibitors. Six patients remain on beta blocker and 4 patients remain on spironolactone. Figure 3 shows the trend of LV ejection fraction and LV end-diastolic z-score following VAD explanation for MFR.

**Table 2:** Clinical Characteristics of Patients Who Died During VAD Support

Pt	Sex	At time of implant		Diagnosis	Type of support	Device type	Days of support	Cause of death/redirection of care
		Age (years)	Weight (kg)					
1	F	0.3	3.1	DCM	BiVAD	PFD	9	Severe infection
2	F	11.4	28.8	DCM	BiVAD	PFD	11	Stroke and severe ARDS
3	M	0.9	7.5	DCM with Barth syndrome	LVAD	PFD	57	Stroke
4	F	16.5	55	Cardiomyopathy with concentric hypertrophy	LVAD	PFD	1	High-risk VAD/multiorgan failure with DIC
5	F	0.7	6.5	DCM due to histiocytoid cardiomyopathy	LVAD	PFD	238	Stroke
6	F	15	84	DCM	LVAD	CFD	18	Stroke

Abbreviations: BiVAD, biventricular assist device; CFD, continuous flow device, DCM, dilated cardiomyopathy; DIC, disseminated intravascular coagulation; LVAD, left ventricular assist device, PFD, pulsatile flow device.

### Main pulmonary artery banding at time of VAD explant

There are 2 patients with DCM who underwent main PA banding at the time of VAD explantation. Neither patient required any intervention on their PA band to dates. The first patient was 9 months old at the time of VAD explant. Due to significant ventricular septal dyskinesia, a PA was applied at time of VAD explant surgery (peak gradient 30-35 mm Hg). This patient is now more than 3 years post-VAD explant. Recent echocardiogram showed good biventricular systolic function with PA band gradient of ∼30 mm Hg (patient number 4).

The second patient was born at 32 weeks of gestation with a weight of 1,570 g with severe LV dysfunction. Due to persistent HF symptoms, the patient underwent PA banding at 2 months of age due to the high risk associated with VAD, as the infant weighed only 3.2 kg at the time. After PA banding, the patient was able to gain some weight but then developed worsening HF symptoms and underwent VAD implantation with PA debanding at the age of 4.5 months and a weight of 4.8 kg. Due to previous history of tolerating PA band and normal RV function at explantation, a PA band was applied at the time of VAD explant to optimize ventricular-ventricular interaction. The patient is now 1.5 years post explant. Recent echocardiogram showed PA band gradient of ∼30 mm Hg (patient number 6).

**Figure 4:**
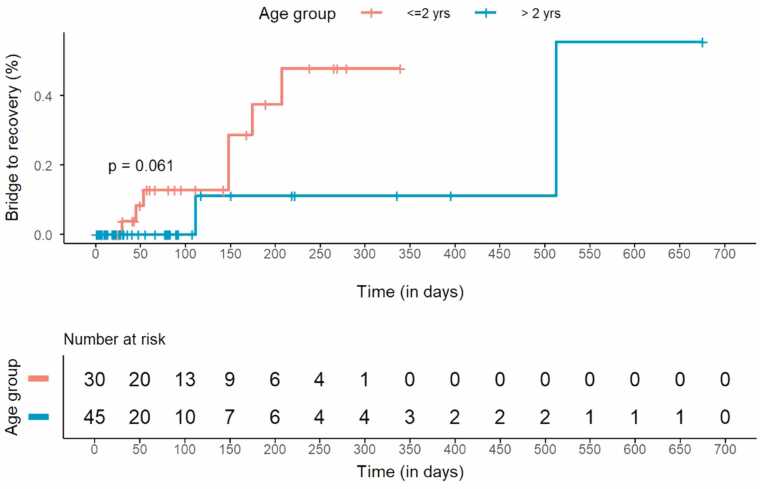
Kaplan-Meier method with log-rank test for comparison of VAD explantation for myocardial functional recovery according to age.

Table 1 shows the univariate analysis between VAD explant cohort for MFR versus nonexplant cohort. The VAD explant cohort was younger (*p* = 0.003) and had longer duration of VAD support (*p* = 0.04) compared to the nonexplant cohort. There was a trend toward higher VAD explantation rate for MFR in children less than 2 years (*p* = 0.061) (Figure 4).

## Discussion

Durable VAD explantation for long-lasting MFR would be the ideal outcome for children with HF requiring MCS. However, there are ongoing challenges with successful VAD explantation as it remains rare in both children and adults.^1^, ^9^, ^10^ In our cohort, 12% of patients who underwent durable VAD support underwent VAD explantation following MFR, with most children doing well post explantation in the ambulatory care setting. Meira et al reported an overall VAD explantation rate of 14.2% with 7.1% in patients with DCM.^11^ Our DCM cohort has a similar incidence of explantation rate in patients with DCM (7.9%). No patient in our explant cohort underwent endomyocardial biopsy before VAD implantation. However, it should be noted that no patient in our study was found to have active myocarditis or acute inflammation at the time of implantation, based on the pathology review of the myocardial core samples taken. In a systemic review of the literature regarding myocardial recovery in children after VAD implant, Rohde et al reported a myocardial recovery rate of 8.7% (81/928) with a heterogeneous population of disease etiologies for HF.^12^

The rationale behind MFR after VAD support is related, in part, to the improvement in hemodynamics and end organ function; but additionally, VAD support provides reduced ventricular end-diastolic pressure, reduction in left atrial pressure, and improved volume state. These hemodynamic changes improve coronary perfusion pressure and cardiopulmonary interactions.^2^, ^14^ When VAD support continues for a longer time frame, it can lead to ventricular reverse remodeling and MFR in a subset of patients.^5^, ^6^, ^7^, ^11^ Reverse cardiac remodeling and MFR should not be considered an “all-or-nothing” phenomenon but represent a continuous spectrum of myocardial changes due to device support.^15^ Previous adult studies showed reduction in ventricular size within 30 to 40 days of VAD support, while improvement in ventricular function is delayed and continued up to 12 to 18 months after implant.^2^, ^5^, ^6^, ^7^ Limited studies in children showed no reduction or early reduction followed by worsening LV end-diastolic size after LVAD implantation.^16^, ^17^ In Canada, wait times for transplantation are long; this affords clinicians the opportunity to continually reassess for MFR while on the transplant waitlist. In our cohort of children who underwent VAD explantation, 70% were on device support for more than 90 days. There is 1 histopathological study that indicates that mechanical unloading by VAD support in children with DCM leads to reverse histological remodeling in the form of reduction in fibrosis and increase in number of cardiomyocytes, the reduction in fibrosis is correlating with younger age.^16^ In our cohort, 3 of 5 (60%) patients who underwent explant for DCM were less than 2 years and all patients less than 2 years of age have remained in HF remission to date.

The strategies to promote MFR following VAD implantation in children have not been well explored. In adults with nonischemic cardiomyopathy, combined LVAD support with GDMT and cardiac function monitoring protocols result in higher rate of MFR and VAD explantation.^5^, ^6^, ^7^ It is important to recognize that pediatric and adult DCM may be distinctly different entities.^18^ The study by Lavine et al showed that pediatric DCM may have less or minimal cardiomyocyte hypertrophy and myocardial fibrosis compared to adult DCM patients, which may also impact the responsiveness of GDMT in children compared to adults.^18^, ^19^ Recently published guidelines/statements from the International Society of Heart and Lung Transplantation recommend the use of GDMT in adults supported on VAD.^20^ The effect of GDMT in children on VAD support has not been well studied to date. The Advanced Cardiac Therapies Improving Outcomes Network has developed a pediatric protocol for weaning off VAD which also incorporates the use of GDMT as part of the process.^21^ Since the inception of our program in 2004, our institutional experience has evolved over time with regards to HF medication use and ongoing monitoring for MFR and optimal LV unloading. Now, we perform periodic assessment for optimal LV unloading and ventricular functional assessment. We also attempt to put all patients on targeted or maximally tolerated doses of GDMT before and post-VAD explantation (Figure 1).

Another important distinction of our study cohort compared to others is the inclusion of patients with congenital heart disease and tachycardia-induced cardiomyopathy. The post surgical repair of ALCAPA, by and large, has excellent results from a transplant-free survival perspective.^22^, ^23^, ^24^ However, it is known that some patients do require MCS post coronary reimplantation in the setting of profound postoperative contractile dysfunction or inability to wean from cardiopulmonary bypass despite an adequate repair. In most instances, VAD support is used as bridge to recovery with encouraging results in previous studies.^25^, ^26^ In our cohort, 3 of the children who underwent VAD explantation had repaired ALCAPA requiring postoperative VAD support; all are doing well with no recurrence of their HF. Of note, there was 1 patient post-ALCAPA repair and VAD support who never showed any substantial MFR, even after 1 year support, and ultimately underwent HT. The effect of early compared to delayed VAD support on ventricular functional recovery in those with an underlying ischemic process needs to be further studied in children. Similarly, 1 patient demonstrated profound cardiac dysfunction after a diagnosis of tachycardia-induced cardiomyopathy. This is another diagnosis that typically shows recovery with resumption of sinus rhythm but can require MCS support in some instances.^27^, ^28^ Both of these disease entities highlight that, though uncommon, the need for end-stage HF therapies and transplantation has been reported in condition that one would normally expect to recovery from.^23^, ^27^

There are several challenges with regard to VAD explantation in children. One of the most important questions that remains unanswered is how to define a “successful explant.” In this study, we demonstrated 1 episode of HF recurrence requiring device reimplantation. However, this patient also managed for a prolonged period away from hospital and sufficient growth that the reimplant was feasible with an intracorporeal CFD. Also, all the diagnostics (invasive and noninvasive) that are performed before device explantation may only show the “short-term” ability to wean from device support. No predictors exist for MFR in the intermediate- or long-term. Decision-making around explantation also needs to include the likelihood of HF recurrence (such as pathogenic genetic mutations and histopathological findings), type of VAD (PFD vs CFD), device complications, and transplant waitlist times which are ultimately reasons why VAD explantation continues to be a rare phenomenon to date. The most recent Pediatric Interagency Registry for Mechanical Circulatory Support (Pedimacs) Report continues to show that nearly 65% of pediatric patients receive a heart transplant less than 6 months after VAD implantation, and thus perhaps we are not waiting long enough for substantial MFR to occur.^1^

This study was able to describe our institutional experience for VAD explantation in a pediatric cohort, which overall demonstrates a positive outcome for most children after explant. VAD support leads to remission of HF via MFR but is likely not “curative” or representative of complete normalization.^2^, ^3^ There is also the risk of recurrence of HF following VAD explantation. The strategies to promote MFR and prevent recurrence of HF, including the concomitant use of GDMT, remain unknown and require further study.

## Conclusion

Sufficient MFR to permit VAD explantation, though uncommon, is possible. Vigilance for evidence of MFR while on VAD support, even while listed for transplant, is prudent. MFR may be more common in children less than 2 years of age with chronic HF on GDMT. Further work is needed to identify the optimal duration of VAD support and the role of GDMT in increasing the likelihood of MFR.

## Limitations

This study is a retrospective study with all the inherent associated limitations. We were unable to identify patients who demonstrated any MFR before being transplanted (i.e., as potential VAD explantations). The small cohort and limited number of explantations in our study are limiting factors for the assessment of the predicators of the MFR. In addition, the approach to assessing and managing patients on VAD support has evolved over the study period as has the use of HF medications while on device support.

## Author contributions

Bhavikkumar Langanecha: conceptualization, data curation, formal analysis, writing—original draft. Osami Honjo: conceptualization, writing—review and editing. Alyssa Power: writing—review and editing. Oshri Zaulan: writing—review and editing. Christoph Haller: writing—review and editing. Kristen George: writing—review and editing. Linda Fazari: writing—review and editing. Andrea Maurich: writing—review and editing. David Chiasson: writing—review and editing. Aamir Jeewa: conceptualization, writing—review and editing, supervision.

## Disclosure statement

The authors declare the following financial interests/personal relationships which may be considered as potential competing interests: Aamir Jeewa reports a relationship with Abbott Industries that includes funding grants. Aamir Jeewa reports a relationship with Berlin Heart Inc that includes consulting or advisory. Aamir Jeewa reports a relationship with Merck & Co, Inc that includes consulting or advisory. The other authors declare that they have no known competing financial interests or personal relationships that could have appeared to influence the work reported in this paper.

This research did not receive any specific grant from funding agencies in the public, commercial, or not-for-profit sectors.

No generative AI or AI-assisted technologies were used in the writing process.
